# Immune Escape Mutants of Highly Pathogenic Avian Influenza H5N1 Selected Using Polyclonal Sera: Identification of Key Amino Acids in the HA Protein

**DOI:** 10.1371/journal.pone.0084628

**Published:** 2014-02-25

**Authors:** Ioannis Sitaras, Donata Kalthoff, Martin Beer, Ben Peeters, Mart C. M. de Jong

**Affiliations:** 1 Quantitative Veterinary Epidemiology, Department of Animal Sciences, Wageningen University, Wageningen, The Netherlands; 2 Department of Virology, Central Veterinary Institute of Wageningen University and Research Centre, Lelystad, The Netherlands; 3 Institute of Diagnostic Virology, Friedrich-Loeffler Institut, Greifswald-Insel Riems, Germany; Thomas Jefferson University, United States of America

## Abstract

Evolution of Avian Influenza (AI) viruses – especially of the Highly Pathogenic Avian Influenza (HPAI) H5N1 subtype – is a major issue for the poultry industry. HPAI H5N1 epidemics are associated with huge economic losses and are sometimes connected to human morbidity and mortality. Vaccination (either as a preventive measure or as a means to control outbreaks) is an approach that splits the scientific community, due to the risk of it being a potential driving force in HPAI evolution through the selection of mutants able to escape vaccination-induced immunity. It is therefore essential to study how mutations are selected due to immune pressure. To this effect, we performed an *in vitro* selection of mutants from HPAI A/turkey/Turkey/1/05 (H5N1), using immune pressure from homologous polyclonal sera. After 42 rounds of selection, we identified 5 amino acid substitutions in the Haemagglutinin (HA) protein, most of which were located in areas of antigenic importance and suspected to be prone to selection pressure. We report that most of the mutations took place early in the selection process. Finally, our antigenic cartography studies showed that the antigenic distance between the selected isolates and their parent strain increased with passage number.

## Introduction

Influenza A viruses belong to the family of orthomyxoviridae. They are negative single-stranded RNA viruses with a segmented genome that comprises 8 genes. Of these, the immunologically most-important are the Haemagglutinin (HA) and Neuraminidase (NA) genes, which encode for the corresponding proteins. There exist 16 different types of HA and 9 of NA, proteins, thus theoretically giving rise to 144 combinations [1]–[3].

Influenza viruses are known to undergo a process called antigenic drift, whereby they continuously change their antigenic and genetic properties. The absence of proof-reading and post-replicative repair mechanisms characteristic of the RNA polymerase of these viruses is an important factor of antigenic drift [4], [5]. The most important outcomes of antigenic drift may be an increased ability of the virus to avoid natural or acquired host-immunity, as well as a possibility of breaching host-range barriers [6]–[8]. Both the HA and the NA proteins are involved in the process of antigenic drift with the HA implicated much more, since it is the main target of neutralising antibodies and is known to accumulate many point mutations in its epitope or antibody-binding regions [9]–[13].

As is the case with many RNA viruses, Avian Influenza (AI) viruses consist of quasi-species. The mutations leading to the antigenic drift discussed above also result in variation in the viruses that occur together in one site of replication. In a quasi-species these different virus genomes act together as one larger genome [14]–[17]. The result is a virus pool that contains variable sequences [4], some of which may in combination offer the virus a competitive advantage by enabling it to adapt to a variety of situations [8], [18], [19].

Highly Pathogenic Avian Influenza (HPAI) H5N1 virus has spread globally and has become endemic in several parts of the world, which is unique for a HPAI strain. Moreover, transmission to humans occurs sporadically with most of the incidents involving poultry workers and handlers and their immediate family members [20]–[22]. Human cases remain sporadic due to the fact that human-to-human transmission of H5N1 is at present inefficient [8], [23]–[25]. Nevertheless, H5N1 viruses are known to continuously undergo antigenic drift as well as gene re-assortment and as such they may become transmissible between humans [26]. For example, recent studies claim to demonstrate airborne transmission of HPAI H5N1 with particular mutations in ferrets [27]–[30].

In the case of humans, world-wide vaccination against seasonal influenza is implemented. Constant screening for new variants ensures that vaccine preparations are up-to-date with currently circulating strains, thus making the vaccines as efficient as possible. In poultry however, vaccination against avian influenza is not as common. Avian influenza is a major problem in most parts of the world, especially South-East Asia, where mainly strains of the H5N1 subtype are endemic. In order to control HPAI outbreaks and thus prevent potential transmission to humans, culling of infected animals as well as pre-emptive culling is the most common method of choice. This has a devastating effect on the economy. A number of countries (i.e. People's Republic of China, Hong Kong SAR, Vietnam, Indonesia, South Korea, Mexico Pakistan and Egypt) are implementing nation-wide vaccination programmes in their fight against avian influenza especially of the H5N1 subtype due to its implications for humans. Nevertheless, none of these countries continuously updates the vaccines used so as to match currently circulating strains based on data from screening programmes, as is the case with human vaccines. In addition, other problems identified in countries where vaccination is used to control infection and transmission of HPAI include – but are not limited to – lack of screening for emerging variants, cutting vaccine doses for economical reasons and inadequately-trained personnel administering the vaccine [31]–[35].

Before vaccination can be used as a strategy against AI, the mechanisms by which selection of escape mutants takes place, as well as, the genetic and – most importantly – antigenic distance necessary for mutants to avoid vaccination-induced immunity must be understood. Although abundant research has been done in escape mutant selection using monoclonal antibodies (MAbs) directed against specific epitopes [36]–[42], research using polyclonal sera to select for escape mutants is very scarce. For example, a study by Lambkin *et al*. [43] selected for escape mutants either by using antisera from mice immunised with inactivated influenza virus or by using a panel of 2–3 MAbs, but in those panels one MAb was consistently present at much higher concentrations compared to the rest. Another study by Archetti and Horsfall [44], selected for antigenic variants of influenza A virus *in ovo* in the presence of heterologous immune sera. While the study was ahead of its time (1950), the technology of gene sequencing and the technique of antigenic cartography were not in place to allow a comprehensive genetic and antigenic characterisation of the mutants selected. Trying to gain an insight in the selection of mutants using MAbs does not fully address the complexity of the issue. On the other hand, research on escape mutant selection in vaccinated poultry is largely limited to field observations, collections and characterisations of isolates originating from some of the countries where a vaccination programme is in place. Although substantial and valuable information has resulted from such research leading to a better understanding of the field situation and its impact in immune escape [45]–[47], such field studies have too many variables, making pinpointing the causes of possible directional selection very difficult.

Genetic characterisation of mutants by sequencing various parts of the genome (with an emphasis on the HA gene) and by identifying the positions and possible functions of various amino acid mutations in the HA protein, is a valuable tool used to elucidate the genetic evolution of a virus. Nevertheless, it does not say much about potential changes in antigenicity of new strains and whether the vaccine used against the original strain would be less effective against these new mutant strains.

To measure possible changes in antigenicity, antibody binding assays are used. The Haemagglutination Inhibition (HI) assay is the golden standard used for decades by laboratories worldwide for surveillance of influenza strains and the characterisation of their antigenic properties [48], [49]. Nevertheless, although the HI assay is a valuable tool and easy to perform, it provides somewhat crude data on the magnitude of the antibody (serum) response against a viral strain but not on the focus and breadth of this response. Furthermore, variations in protocols and origin of red blood cells, coupled with the wide spacing of the dilutions used in these assays, may make results from extensive HI databases on strains that have been tested worldwide difficult to interpret. In addition, the multitude of raw data generated by such HI databases makes them very difficult to be visualised as a whole. It is precisely for these reasons that a new computational technique called antigenic cartography was developed and is now used widely by the WHO [50]–[53]. Antigenic maps involve the construction of distance matrices between viral isolates as measured by the HI assay, and the plotting of these distances in a way that is analogous to a geographical map. The further the distance between two strains, the greater the antigenic differences between them [52]. Antigenic cartography not only allows for the simple and immediate visualisation of HI data, but also gives an insight into virus evolutionary dynamics by allowing us to compare genetic changes (as reflected by sequencing and construction of phylogenetic trees) to phenotypic changes (as reflected by antigenic distances) [12]. Furthermore, combining genetic and antigenic characterisation can provide insights on which particular areas in the HA protein are sensitive to directional selection, and which mutations can alter the antigenicity of strains. Finally, by having a picture of the antigenic evolution of a virus, antigenic cartography can help us decide whether vaccine updating is necessary [50]–[53].

In this study, we aim to study the evolution of AI viruses by repeated *in vitro* exposure to targeted pressure from polyclonal immune sera directed against the HA of HPAI H5N1 A/turkey/Turkey/1/2005. We characterised the resulting mutants by identifying the nucleotide and amino acid substitutions in the HA gene and protein respectively and by examining and quantifying whether these substitutions resulted in genetic and antigenic differences between the mutants and the parent strain. Furthermore, we broadened our antigenic characterisation by including escape mutants previously described [54] and by comparing these mutants to our own.

## Materials and Methods

### Ethics Statement

All animal experiments complied fully with Dutch Law and were reviewed by the Dierexperimenten Commissie (DEC) Animal Sciences Group, Lelystad (animal experiments ethical committee) prior to being carried out. The animal experiments necessary to produce the antisera as described below, were approved by the committee: permit numbers 2010123 and 2011086. Provisions were made in the protocols to ameliorate animal suffering, such as regular monitoring of animal facilities, healthcare of animals and termination of animals by means of cervical dislocation at the end of the experiment (or during the experiment if serious health problems were noticed).

All experiments described in this paper were performed within the High Containment Unit, in Bio-Safety Level 3+ (BSL3+) facilities at the Central Veterinary Institute of Wageningen UR.

Experiments regarding selection of mutants and genetic characterisation refer to CVI mutants only. Both the CVI mutants as described here and the FLI mutants described in Höper, *et al*., [51] were used for antigenic characterisation experiments such as the generation of sera, HI assays and the construction of the antigenic maps.

### Preparation of H5N1 virus stock

HPAI H5N1 influenza strain A/turkey/Turkey/1/2005 (Clade 2.2.1) was used as a parent strain (from now on abbreviated to H5N1 t/T). A stock was made by passaging the virus once in Specific Pathogen-Free (SPF) 9-day-old Embryonated Chicken Eggs (ECEs) obtained from Charles River Avian Vaccine Services. Standard Haemagglutination Assays (HA) were performed on the allantoic fluids of individual ECEs and the samples with the same HA titres were pooled. Another HA assay was performed to determine the HA titre of the pooled samples. The pooled stock had a HA titre of 64 Haemagglutination Units (HAU) and a Tissue Infectious Dose 50 (TCID_50_) of 10^8.3^/mL. All TCID_50_ assays were performed according to standard protocols and using Madin Darby Canine Kidney (MDCK) cells.

### Origin of Sera for Selection Experiments

The sera used for our selection experiments were obtained from SPF animals vaccinated against a genetically-modified (GM) H5N7 strain, with the HA gene originating from A/turkey/Turkey/1/2005, inserted into the backbone of the HPAI H7N7 Dutch outbreak virus A/chicken/Netherlands/6215857/2003 (Peeters *et al*., Unpublished Results). Briefly, animals were vaccinated with 0.1% paraformaldehyde-inactivated GM H5N7 in the presence of Stimune adjuvant (Prionics) at a 4∶5 (v/v) inactivated virus to Stimune ratio. Vaccination volume and route were 0.5 mL intra-muscularly (leg muscle) respectively. The HA protein content of the vaccine was 0.6 µg/0.5 mL. Twenty-one days post-vaccination, the animals were vaccinated for a second time with the same vaccine dose. Sera were collected 11 days after second vaccination took place. The HI titre of each serum was determined and sera with HI titres 2^7.5^–2^10.5^ (181–1448 units) were pooled. The HI titre of the pooled sera used in the series of experiments described in this paper was 2^9.5^ (i.e. 724 units).

### Determination of optimum serum dilution for selection of mutants

Dilutions of sera in Dulbecco's Phosphate Buffered Saline (DPBS) ranging from 1∶100 to 1∶1600 were made. Each serum dilution was incubated for 2 h at 37 °C with an equal volume of 2^4^ (16) HAU of the parent strain. After the incubation, TCID_50_ assays were performed on virus incubated with each dilution. An optimum serum dilution for starting the selection experiments was defined as the dilution that resulted in a 1000× titre decrease from the original TCID_50_ titre. As such, it was found that a 400× serum dilution should be used for selection of mutants.

### Selection of Mutants

Selection of mutants took place *in vitro*. In detail, for each subsequent round of selection, 16 HAU of virus from the immediately previous selection round (starting with the parent strain) were incubated with an equal volume of H5 homologous sera (starting with a 400× dilution) for 2 h at 37°C. As a control, 16 HAU of virus isolates from the same selection round were incubated with the same dilution of non-immune sera (i.e. sera derived from SPF chicken) as the one used for the selection with immune sera. After the incubation, the control isolates (i.e. virus incubated with non-immune sera) were diluted 1000× in DPBS in order to reduce the number of live virus particles, so as to resemble the reduction that had taken place in the selection isolates. Passages took place in three 9- to 11-day-old ECEs inoculated with 200 µL of the selection isolate and diluted control isolates. The inoculated ECEs were incubated at 37°C, 5% CO_2_ and were checked for embryo deaths regularly. Dead embryos were removed from the incubator, time of death was recorded and the allantoic fluid was harvested. HA assays were performed on the allantoic fluid of each ECE and the ones with the same or similar (within 1 log_2_ difference) HA titre were pooled, had their HA titre re-checked and used for the next selection round. The same procedure was followed for 20 passages. After passage 20, serum pressure on the virus isolates was gradually increased by decreasing the dilution of sera the virus isolates were incubated with until the last passage (passage 42). Criteria for decreasing serum dilutions were an increase in the HA titre of the virus isolated from the allantoic fluid compared to the input titre (16 HAU) and an earlier time of death of the embryos. As a consequence of the continuous decrease in serum dilutions, a 1∶5 dilution was used in the last rounds of selection, marking a difference of 80× compared to the 1∶400 used in the first 20 selection rounds. For both the selection and control isolates the same serum dilutions were used throughout.

### RNA Isolation/DNA Amplification

Viral RNA was isolated from the allantoic fluid of every 5^th^ passage (both for the selection and control isolates), using the High Pure Viral RNA Kit (Roche) according to the manufacturer's instructions. First strand cDNA synthesis (RT-PCR) was carried out on the isolated RNA material, using SuperScript® III, First-Strand Synthesis SuperMix (Invitrogen) and Uni12, a universal RT primer (primer sequence available upon request). Amplification of the cDNA was done using the Expand High Fidelity PCR System (dNTPack) from Roche and one pair of primers amplifying the entire HA gene (primer sequences available upon request).

The amplified HA was checked in a 1% agarose gel and the PCR product was purified using the High Pure PCR Product Purification Kit (Roche) as per manufacturer's instructions. The DNA content of the purified PCR product was measured spectrophotometrically (NanoDrop™ 1000, Thermo Scientific Inc.).

### Sequencing of the HA of mutants

Sequencing PCR on the purified PCR product took place using 4 pairs of forward and reverse primers spanning the entire length of the gene with overlapping regions (primer sequences available upon request).

DNA from sequencing PCR was precipitated and samples were prepared for sequencing analysis, using the 3130 Genetic Analyser (Hitachi/Applied Biosystems).

The SeqMan™ software from DNASTAR Lasergene® 10 Core Suite was used to align, edit and compare the nucleotide sequences of the passage isolates to the sequence of the parent strain. Nucleotide sequences were translated into amino acid sequences and aligned using the MegAlign™ software of the same suite, and the positions of the mutations were noted.

### Erythrocyte Binding Assays

Erythrocyte binding assays were performed to evaluate whether there exists a difference between the H5N1 t/T parent strain and the latest mutant, CVI Passage 42 (from now on abbreviated to CVI P42) in their ability to bind to chicken erythrocytes. The assays were performed in triplicate. To avoid any NA activity from taking place, which would result in release of virus from the erythrocytes, the assay was performed on ice for the entire duration. Time points (0, 5, 10, 20, 30 and 40 minutes) were introduced in order to evaluate the kinetics of virus binding to erythrocytes. Finally, a ratio of 1000∶1 erythrocytes:virus particles was used. Both strains were diluted to 1.75×10^4^ virus particles/200 µL and incubated on ice with an equal volume of 1% erythrocytes (1.75×10^7^) from SPF chicken at the indicated time points. As a control, the same amount and volume of viruses were added to 200 µL of DPBS at time point 0 minutes. Immediately after the incubation, the samples were centrifuged gently at 1500 rpm in a pre-cooled centrifuge to precipitate the erythrocytes, 200 µL of supernatant were removed from each time point and viral RNA was isolated with the MagNA Pure 96 system (Roche Applied Sciences), using the MagNA Pure DNA and Viral RNA Small Volume kit (Roche Applied Sciences) as per manufacturer's instructions. Quantitative R-RT-PCR (qPCR) using the Mx-3005P (Stratagene) was used to quantify viral copies after 45 cycles of amplification. The qPCR protocol and the AI probe used were optimised to be specific to the highly conserved gene encoding for the M protein of influenza A viruses. A standard curve comprising of the H5N1 t/T strain was prepared and used to analyse the data. The results from the qPCR were analysed using the MxPro software (Stratagene). Since the results from the triplicate measurements were highly reproducible (data not shown), the mean of the three measurements for each time point was used.

### Inactivation of Viruses

All strains were inactivated by incubating virus stocks with 0.02% paraformaldehyde (Merck) for 16 h at 37°C. HA assays were performed on the inactivated viruses before and after inactivation to note HA titres. Complete inactivation was verified by two passages in ECEs lasting 7 days each, followed by HA assays at the end of each passage to check for the presence of live virus. The inactivation was considered successful when the HA assays at the end of each passage were negative for each strain.

The inactivated strains had their HA titres standardised to 2^6^ (64) HAU by diluting them with negative allantoic fluid (i.e. allantoic fluid originating from SPF ECEs).

### Generation of Hyper-Immune Sera for Antigenic Characterisation

In order to examine the antigenic differences between passage isolates and their respective parent strains by means of constructing antigenic maps, we generated sera in chickens, against a number of selected strains.

In detail, we have raised sera against the parent strain (H5N1 t/T) and the CVI P20 and CVI P42 isolates. In addition to our isolates, sera were also raised against the FLI parent strain (HPAI H5N1 A/Cygnus cygnus/Germany/R65/2006, from now on abbreviated to H5N1 R65) and selected mutants (FLI P18esc and FLI P30esc). These mutants are described in detail in Höper *et al*. [51].

For reasons of comparison and in addition to the parent strains and mutants of both groups, sera were also raised against two other HPAI H5 strains which are known to be antigenically different from the parent strains used by both CVI and FLI. These were A/chicken/Pennsylvania/1370/1983 (H5N2) and A/tern/South Africa/1961 (H5N3).

A complete list of the strains used to produce sera in chickens, is shown in Table 1.

**Table 1:** List of strains used for antigenic cartography studies.

Strain Name	Accession Number	Origin
HPAI H5N1 A/turkey/Turkey/1/2005	EF619980	CVI
CVI P20	KF042152	CVI
CVI P42	KF042153	CVI
HPAI H5N2 A/chicken/Pennsylvania/1370/1983	GU052771	CVI
HPAI H5N3 A/tern/South Africa/1961	GU052822	CVI
HPAI H5N1 A/cygnus cygnus/Germany/R65/2006	EPI309750	FLI
FLI P18esc	EPI287212	FLI
FLI P30esc	EPI287220	FLI

Strains against which sera were raised for purposes of antigenic cartography. P stands for Passage. In the interests of continuity, the same naming for the FLI strains mentioned in [54] was kept.

All SPF animals used in our experiments originated from SPF ECEs that were hatched in our HCU facilities. Two 6-week-old SPF chickens per strain were each vaccinated intra-muscularly with 0.5 mL of 64 HAU inactivated virus in the presence of adjuvant (Stimune, Prionics) at a 4∶5 (v/v) inactivated virus to Stimune ratio. The animals were housed together and were inspected twice a day. Three weeks post-vaccination, the animals were bled, sera were collected and inactivated at 56°C for 50 minutes. The inactivated sera were stored at −20°C.

### HI Assays

The titres of the inactivated sera were determined in HI assays [48], [49], in which 8 HAU of each strain were used as an antigen for each one of the sera that were collected during the animal experiments (i.e. homologous and heterologous sera). The assays took place in duplicate. All 8 strains used for the generation of hyper-immune sera were used in the HI assays. In addition, the intermediate CVI passage isolates (CVI P5, P10, P15, P25, P30, P35 and P40) were also used, in order to evaluate which isolates are more suitable for use in antigenic cartography studies.

### Construction of Virus Antigenic Map

Our HI dataset consisted of 16 sera that were raised against 8 strains (2 animals/strain). In addition, each serum measurement was performed in duplicate.

To construct the virus antigenic map, we first averaged the duplicate HI titres for each serum and converted the values into log_2_ values. The result was a 16×8 matrix.

We then calculated the difference in log_2_ titres between the homologous (i.e. sera raised against the same strain) and heterologous (i.e. sera raised against the remaining 7 strains) scenarios. We standardised the differences to be ≥0, thus any negative values were treated as 0. The result was a 16×8 matrix which depicts in each row the differences in HI titres between each one of our 8 strains against each of the 16 sera.

The matrix was standardised so that the average of each virus against each of the 16 sera would be 0 and the SD would be 1.

From this 8×16 matrix (M) with the standardised differences, the distances between strains were calculated in Mathematica® (Version 8, Wolfram Research, Inc.) by applying the following formula,
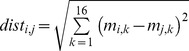
where m is a matrix element of matrix M.

From the resulting symmetrical 8×8 distance matrix, we are able to compare the distances between any set of three strains by extracting the 3×3 distance sub-matrices of these strains and solving the distance equations to obtain x, y coordinates for each strain. As a result, this distance matrix can be presented on a 2-dimensional plane by plotting triangles that share one edge and thus conserve the real distances between the viruses. Hence, in such a plot distances between all viruses connected by a triangle represent the exact distance as found in the distance matrix but this is not true for the viruses that do not share a triangle.

### Construction of Sera Antigenic Map

To construct an antigenic map of the sera, the same procedure was followed, but applying modifications to show the position of each serum from each strain, relative to the sera of the other strains. Our starting point was thus an 8×16 matrix. By constructing such a map, we also obtain information on the differences in the immune responses of individual chicken.

Thus, the distance formula is modified as follows:
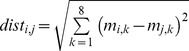



## Results

### Genetic Characterisation of Passage Isolates by HA Sequencing

Mutants were selected in the presence of homologous immune sera. Serum dilutions were gradually lowered after passage 20 in order to increase selection pressure. An overview of the serum dilutions used for each passage appears in Table S1 in File S1.

Isolates from every 5^th^ passage (both from the selection and control lines) were selected for sequencing of the HA gene. Nucleotide and amino acid substitutions were noted. The amino acid numbering reported here refers to the reference H5N1 strain A/goose/Guangdong/1/1996.

**Figure 1:**
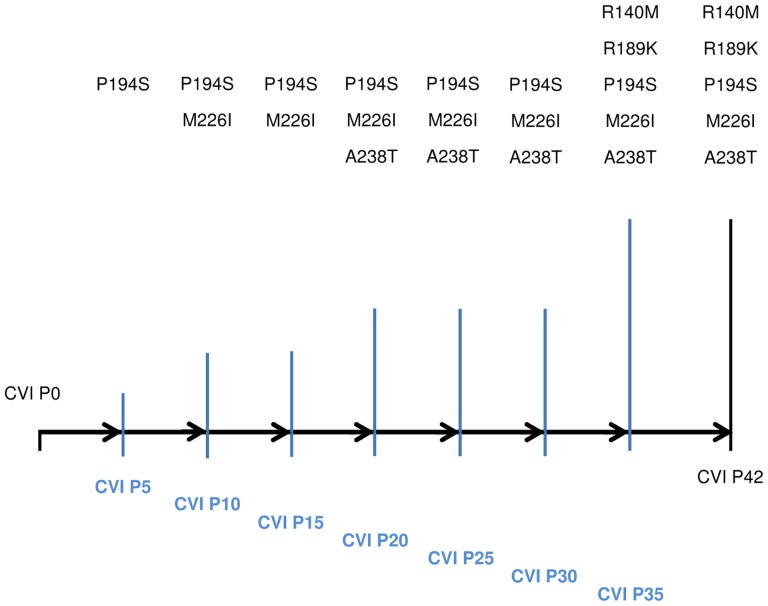
Amino acid mutations found in the HA of CVI passage isolates. Schematic representation of the mutations found in CVI passage isolates. The number of amino acid mutations increases with passage number and serum pressure. First mutations are found as early as Passage 5 and are carried over to later passages. Only two additional mutations were found between Passage 20 and Passage 42, indicating that most of the selection takes place in early passages. Black horizontal bar represents the progress of the passages. Blue vertical bars represent the time points where HA sequencing took place and their height represents the number of amino acid mutations found at each time point.

By the end of the selection (CVI P42), 5 amino acid mutations were identified. Crucially, no amino acid substitutions were found in the HA of the control passages. An overview of the amino acid mutations found in the HA protein of our passage isolates as well as the points in the selection process where these mutations were identified are shown in Figure 1. CVI P20 and CVI P42 HA nucleotide sequences were submitted to GenBank (Accession Nos: KF042152 and KF042153 respectively).

Table 2 provides an overview of the HA mutations found in CVI P42 and the antigenic sites (where identified) they fall into. Interestingly, most of the mutations are located in previously-identified Antigenic Sites (AS), and the Receptor Binding Site (RBS). The exception is A254T, which falls in a region not previously connected with antigenicity. In Table 2 a comparison of the A/goose/Guangdong/1/1996 numbering with the numbering of the two parent strains used by CVI and FLI (H5N1 t/T and H5N1 R65 respectively) is also shown.

**Table 2:** Summary of amino acid mutations found in the HA protein of CVI P42, and their locations in respect to antigenically-relevant regions.

Isolate	H5 A/turkey/Turkey/1/05 Numbering	H5 A/goose/Guangdong/1/96 Numbering	Location
CVI P42	R156M	R140M	AS (Epitope B), PS Site, AP
	R205K	R189K	AS (RBS), PS Site, AP
	**P210S**	**P194S**	AP
	**M242I**	**M226I**	AS (Epitope D)
	A254T	A238T	NA

Amino acid numbers are shown according to A/turkey/Turkey/1/05 (H5N1) and the reference strain A/goose/Guangdong/1/96 (H5N1) numbering (the latter numbering is used throughout the report unless stated otherwise). Mutations found in common between CVI and FLI isolates are shown in bold. The locations of amino acid substitutions in respect to antigenically relevant regions – according to Duvvuri, *et al*., (2009) – are shown in the last column (where available). AS: Antigenic Site; PS Site: Positive Selection Site; AP: Antigenic Peptide; RBS: Receptor Binding Site; NA: Not Assigned a function.

### Antigenic Characterisation of Strains and Sera

In order to examine the antigenic differences between each strain (i.e. parent strains and CVI and FLI variant strains), a series of HI assays were performed, in which all sera obtained from animals vaccinated against each strain were cross-checked against each one of the strains. In addition, the intermediate CVI passage isolates were also used in the assays. Regarding the CVI isolates, based on the HI data and the construction of antigenic distance matrices (Tables S4 and S5 respectively in File S1), it was decided to show the CVI P20 and CVI P42 isolates in the antigenic maps of strains and sera. This was because CVI P42 was found to be the most distant isolate compared not only to the H5N1 t/T but also the rest of the CVI isolates. In other words, to fully evaluate the magnitude of the distance of any strain, its distances to all other strains have to be taken into consideration. For example, although the distance between H5N1 t/T and CVI P40 appears to be slightly larger than the distance between H5N1 t/T and CVI P42 (log_2_2.61 and log_2_2.17 respectively), the distance between CVI P40 and CVI P42 is also large (log_2_1.38). This holds true for any strain compared to CVI P42. Figure S1 in File S1 shows the distances of all strains compared to H5N1 t/T and CVI P42. Figure S1 in File S1 provides a map with coordinates (similar to latitude and longitude in a geographical map) where each CVI strain is positioned according to its distance from these two strains. From Figure S1 in File S1 it becomes clear that the antigenic distance between CVI P42 and – say – the cluster of CVI P5, P10 and P15 is larger than the distance of CVI P40 to the same cluster of isolates, thus indicating the greater antigenic distance of CVI P42 across the whole spectrum of CVI strains. CVI P20 was chosen not only because of its antigenic distance to other strains but also because it represented a turning point in our selection process (from a steady immune pressure up to CVI P20 to a progressively increased pressure from there on). Due to these results, the 8 strains used to raise the hyper-immune sera were cross-checked against 2×16 sera (since two animals were vaccinated against each strain and each serum was checked in duplicate).

The data obtained from the HI assays demonstrated that on occasions there existed large differences in the HI titres of sera obtained from the two individual chickens vaccinated against the same strain. In other words, animals in the same vaccination group showed a considerable variation in the individual immune response to the vaccine. In addition, because of the differences in immune response observed occasionally between animals of the same groups, the HI titres obtained from cross-checking the sera from each group of two chickens against each one of the strains show a different pattern of response in the two animals belonging to the same vaccination group. However, the response pattern of each serum is consistent across the strain spectrum. Furthermore, in our analysis for the construction of the antigenic maps we have averaged over these differences. Table 3 provides an overview of the HI data.

**Table 3:** HI data of strains and sera used for construction of antigenic maps.

Sera		Strains							
Vaccine Groups	Animal Numbers	CVI			FLI			Other	
		H5N1 t/T	P20	P42	H5N1 R65	P18esc	P30esc	H5N2	H5N3
**H5N1 t/T**	376	256	128	256	256	128	64	32	64
		128	128	512	128	128	64	32	64
	377	1024	512	1024	1024	1024	128	64	64
		1024	512	1024	1024	1024	128	64	128
**CVI P20**	378	2048	2048	1024	1024	2048	2048	256	256
		1024	1024	1024	1024	2048	1024	256	256
	379	512	256	512	256	512	128	64	64
		256	256	512	512	256	128	64	64
**CVI P42**	742	512	256	1024	512	128	128	64	64
		256	512	1024	256	128	256	64	64
	743	512	512	1024	256	256	128	256	128
		256	256	1024	256	256	128	256	128
**H5N1 R65**	380	512	256	256	512	256	32	32	128
		512	128	256	1024	512	64	64	128
	381	256	256	256	256	64	32	256	32
		256	256	256	256	128	32	256	32
**FLI P18esc**	382	128	64	128	128	512	128	32	64
		128	64	128	128	512	128	32	64
	383	512	256	256	512	1024	256	64	128
		256	256	256	512	1024	256	64	128
**FLI P30esc**	384	64	32	64	64	64	128	4	16
		32	64	128	64	64	128	4	16
	385	128	64	256	128	128	128	16	32
		128	128	256	128	128	128	16	32
**H5N2**	386	64	16	32	64	32	8	1024	64
		64	32	32	64	32	8	1024	64
	387	64	64	64	64	128	64	1024	64
		64	64	64	64	128	64	1024	64
**H5N3**	744	512	128	128	512	1024	128	256	2048
		512	128	128	256	1024	128	256	2048
	745	64	32	128	64	128	64	64	512
		64	32	128	64	64	64	64	1024

In total, 16 sera were raised against 8 strains (2 sera/strain). Each serum (represented by animal number) was checked in duplicate against each one of the strains to record antigenic differences between them.

The calculated distance matrices for viruses and sera are shown in Tables S2 and S3 respectively in File S1. All distances are log_2_ values, therefore, a distance of 2 represents a difference value of 4 units in the HI test. Table S5 in File S1 shows the distance matrix for all CVI isolates.

**Figure 2:**
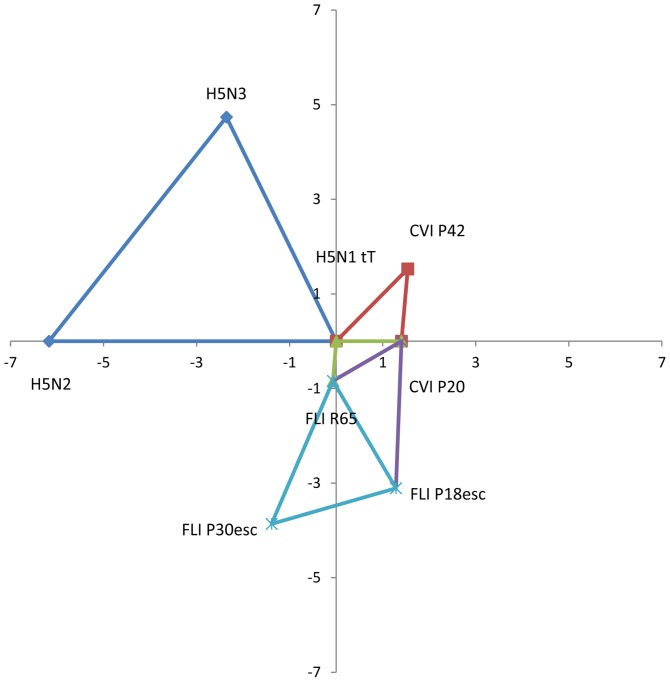
Antigenic map of sets of strains used in this study. Antigenic map showing the standardized real distances between different sets of three strains. Distances are in log_2_ values. Antigenic distance between H5N1 t/T and CVI P20 or CVI P42 is 1.4 and 2.17 respectively. Distance between H5N1 R65 and FLI P18esc or FLI P30esc is 2.64 and 3.3 respectively. Both parent strains (H5N1 t/T and H5N1 R65) are antigenically similar (distance of 0.84). Both parent strains are very antigenically distant to the H5N2 and H5N3 strains used in the study. In detail, the distance between H5N1 t/T and H5N2 or H5N3 is 6.17 and 5.29 respectively, while the distance between H5N1 R65 and H5N2 or H5N3 is 6.34 and 5.42 respectively (all distances can be found in Table S2 in File S1).

**Figure 3:**
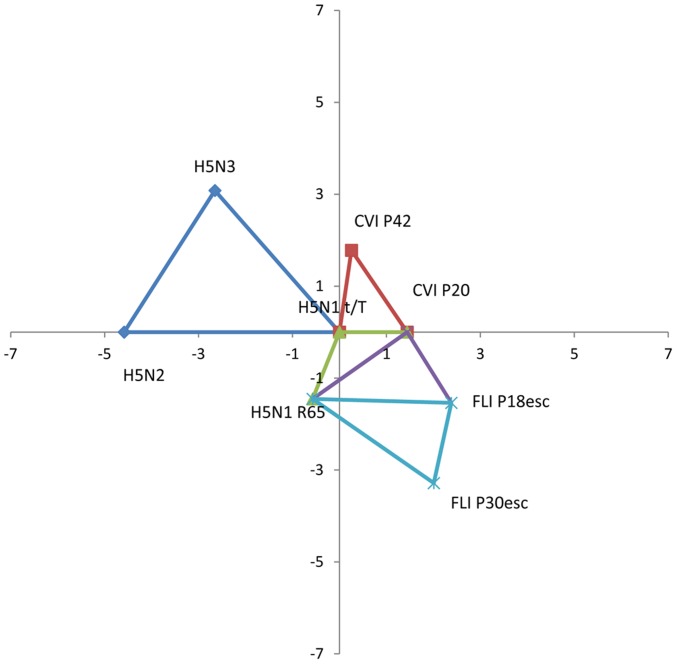
Antigenic map of sets of sera raised against the strains used in this study. Antigenic map showing the standardized real distances between different sets of three sera. Distances are in log_2_ values. Antigenic distance between sera from H5N1 t/T and CVI P20 or CVI P42 is 1.44 and 1.8 respectively. Distance between sera from H5N1 R65 and FLI P18esc or FLI P30esc is 2.95 and 3.16 respectively. Sera raised against the parent strains (H5N1 t/T and H5N1 R65) have a distance of 1.56. Sera raised against H5N1 t/T and H5N1 R65 are very antigenically distant to sera raised against the H5N2 and H5N3 strains used in the study. In detail, the distance between sera raised against H5N1 t/T and H5N2 or H5N3 is 4.58 and 4.07 respectively, while the distance between sera raised against H5N1 R65 and H5N2 or H5N3 is 3.71 and 4.14 respectively (all distances can be found in Table S3 in File S1).

The antigenic maps showing the standardised real differences (log_2_ titres) between different subsets of three strains or sera are shown in Figures 2 and 3, respectively.

According to the antigenic map of the strains (Figure 2), the antigenic distance between the CVI parent strain (H5N1 t/T) and CVI P20 or CVI P42 isolates is 1.4 and 2.17 respectively. Likewise, the distance between the FLI parent strain (H5N1 R65) and the FLI P18esc or FLI P30esc is 2.64 and 3.3 respectively. Both parent strains are antigenically similar, with a 0.84 distance between them. As expected, the distance between H5N1 t/T and the H5N2 or H5N3 viruses is very large (6.17 and 5.29 respectively). Similar results are obtained when comparing the H5N1 R65 to the same two viruses (6.34 and 5.42 respectively).

From the antigenic map of the sera (Figure 3) a similar picture to the map of the strains can be seen. Explanatory, the distance between sera generated against H5N1 t/T and the CVI P20 or CVI P42 isolates is 1.44 and 1.8 respectively and between H5N1 R65 and FLI P18esc or FLI P30esc is 2.95 and 3.16 respectively. Distance of sera generated against H5N1 t/T and H5N1 R65 is 1.56. Finally, the antigenic distance between sera generated against H5N1 t/T and the two reference strains (H5N2 and H5N3) is 4.58 and 4.07 respectively. Similar results are obtained when comparing the antigenic distance of the last two strains against H5N1 R65 (3.71 and 4.14 respectively).

### Comparison of Erythrocyte Binding Ability between H5N1 t/T and CVI P42

Erythrocyte binding assays were performed in a timed manner using the H5N1 t/T and CVI P42 strains. The results showed no difference in the ability to bind to erythrocytes between the two strains. In detail, the kinetics of virus binding were comparable for both strains (from log_10_4.383 to log_10_3.199 for H5N1 t/T and from log_10_4.912 to log_10_4.180 for CVI P42). Similar reductions in virus quantity were observed during the intermediate time points in the assay. An interesting observation that emerged from the timed erythrocyte binding assays was that binding of virus to erythrocytes appeared to be a rapid process in that most of the binding appeared to take place very early in the incubation period (i.e. within the first 10–15 minutes). After this time period, the number of virus particles present in the supernatant appeared to plateau. The results of the erythrocyte binding assays are shown in Figure S2 in File S1.

## Discussion

In this report we studied the effect of immune pressure (by means of polyclonal sera directed against the HA protein) on the evolution and mutant selection of HPAI H5N1 viruses, as well as examined the genetic and antigenic differences of the mutants resulting from this pressure.

The results from the sequencing of the HA gene of our mutants lead to some interesting observations.

Sequencing analysis of passage isolates showed that most of the selection takes place early on, with the first amino acid mutation identified as early as CVI P5. In addition, amino acid mutations accumulate with increased passage number. In detail, CVI P5 demonstrated one amino acid mutation in the HA protein (P194S), while in CVI P10 and P15 a second amino acid mutation is present (M226I). CVI P20 has a third amino acid mutation (A238T). Thus, by keeping the serum pressure constant (1∶400 dilution) for the first 20 passages, three amino acid mutations were identified in the HA protein. After passage 20, the serum pressure was increased and this resulted in the selection of two more amino acid mutations, namely R140M and R189K from CVI P35 onwards. These isolates were selected with a 1∶5 dilution of immune polyclonal sera. These results also show that amino acid mutations found in early passages are carried over to later ones, suggesting they are associated with an increased ability to withstand immune serum pressure as a result of the selection process and are not random mutations.

In addition, the fact that no amino acid substitutions were found in the CVI control isolates indicates that the mutations found in the isolates selected under immune pressure cannot be attributed to events such as adaptation to the culture system or random drift due to replication errors.

Finally, due to the way our selection was carried out, we can exclude the possibility that the CVI mutants were selected due to their increased ability to bind to cells as suggested by Hensley *et al*., [55], since the CVI selection process was cell-free. Indeed, erythrocyte binding assays showed that CVI P42 does not exhibit an increased ability in binding to erythrocytes compared to H5N1 t/T (Figure S2 in File S1). This finding adds credibility to the notion that the mutations found in the CVI isolates can indeed be attributed to directional selection resulting from the polyclonal sera used in this study. Furthermore, if the selection was driven by differences in cell-receptor binding between our parent strain and mutants and not by serum pressure, then some or all of the mutations found in the CVI isolates selected under immune pressure should have also been found in the CVI control isolates, which was not the case, since no mutations were found in these isolates. This is a further indication that we selected for viruses with lower avidity for antibody binding but not necessarily with lower avidity for cell-receptor binding. The results from the extensive HI experiments confirm the difference in antibody binding avidity between the parent strains and the mutants and between the mutants themselves. Nevertheless, it should be pointed out that despite the fact that our results show no difference in binding to erythrocytes between the parent strain and CVI P42, more sophisticated methods may be used to identify subtle differences in receptor-binding avidity or receptor specificity. Furthermore, whether any of the mutations found in our isolates – singly or in combination – would have an effect on cell-receptor binding avidity is a matter of future investigation.

A comparison between the amino acid mutations found in CVI and FLI isolates is shown in Table 4. Intriguingly, two of the amino acid mutations found in early CVI passage isolates (P194S and M226I) were also found in early FLI passage isolates (starting from FLI P18esc). These mutations are shown in bold in Table 4. Considering that isolates between the two groups were selected independently by different types of immune pressure (homologous sera in the case of CVI as opposed to heterologous sera in FLI) and in two different culture systems (ECEs in CVI as opposed to MDCK cells in FLI) and adding the fact that these two mutations were selected early in the procedure, it would appear that the P194S and M226I mutations are particularly significant in H5N1 viruses stemming from the Guangdong lineage when adapting to immune pressure. All other mutations reported in Table 4 are unique to each group.

**Table 4:** Summary and Comparison of Amino Acid Mutations found in the HA protein of CVI and FLI Isolates.

CVI P5	CVI P10	CVI P15	CVI 20	CVI P42	FLI P18esc	FLI P30esc
						N72D
						K119N
				R140M		
						S141P
					K152E	K152E
				R189K		
**P194S**	**P194S**	**P194S**	**P194S**	**P194S**	**P194S**	**P194S**
						T195A
	**M226I**	**M226I**	**M226I**	**M226I**	**M226I**	**M226I**
			A238T	A238T		
						Y271H

Mutations in bold indicate mutations found by both groups. Numbers refer to the A/goose/Guangdong/1/1996 (Influenza Research Database accession number AF144305) HA amino acid sequence.

Phylogenetic analysis using the Maximum Likelihood method based on the Tamura-Nei model [56] showed that both CVI P20 and CVI P42 branched out from their parent strain (data not shown). A phylogenetic tree with the same strains constructed using the Neighbour-Joining method [57] showed similar results (data not shown).

These results confirm the genetic divergence of our mutants. The same applies for the FLI P18esc and FLI P30esc isolates. As expected, all isolates are genetically different from the H5N2 and H5N3 strains, more so than from the ancestral A/goose/Guangdong/1/96 strain.

In accordance with other reports [38], [46], [58]–[60], most of the mutations found in the CVI P20 and P42 isolates are located within known antigenic sites and sites known to be influenced by selection pressure (Table 2). A notable exception is mutation A238T, which is located in a position not yet assigned an antigenic function.

Due to the positioning of our mutations it is not surprising that the same amino acid mutations found in our isolates or different amino acid mutations found in the same positions have been associated with a wide variety of functions ranging from antigenic drift, genetic diversity and even increased recognition of human type Sialic Acid (SA) α2,6 receptors. An overview of the suggested functions and implications of these mutations follows.

Two of the positions where mutations were found in the CVI mutants (namely 140 and 226) have been identified in viruses isolated from vaccinated chickens in Egypt, with position 140 possibly being implicated in immune escape. In detail, research on avian isolates circulating in Egypt (where vaccination is applied both to commercial and backyard poultry, albeit with variable degrees of coverage ranging from around 50% in commercial poultry to 1–25% in household flocks), has shown that a particular 2008 H5N1 isolate is genetically and antigenically different from 2006–2007 isolates [46]. In total, 12 amino acid substitutions were found in the 2008 isolate, 11 of which occurred in the RBS, in locations that are proximal to the functional regions, represented by the 190 α-helix, the 130-loop and the 220-loop. The authors found that mutations in 5 key amino acid positions (including position 140) resulted in reduced HI titres (when compared to the 2007 H5N1 strain not carrying these mutations) and that this effect was produced by substitutions in position 140, either singly or in combination with other substitutions. As a consequence, mutations in these positions appear to be primarily involved in antigenic drift and progressive accumulation of mutations at multiple antigenic sites of the HA1 molecule can enhance antigenic drift, leading to an increased chance of escaping immune responses [46], [59]. In agreement with previous reports [38], [58], [60], [61], an amino acid mutation in position 226 does not appear to contribute to antigenic drift, since this area of the protein is not exposed to the surface of the HA antigen [46]. Nevertheless, the fact that a mutation in this position (M226I) was identified in both the CVI and FLI isolates could allow us to speculate that it has an effect in virus survival and adaptation to immune pressure.

Additional research on Egyptian isolates reported a number of mutations – some of which in the same positions identified in the CVI isolates and some (P194S, M226I and A238T) being identical to the ones found in CVI P10 throughout CVI P42 – resulting in two different sub-lineages of viruses, one falling in the 2.2.1 clade and the other one being a variant group comprising a new 2.2.1.1 sub-clade. The viruses belonging to these groups were reported to have undergone significant antigenic drift and their HAs not to be cross-immunogenic with the HAs of other sub-lineages. The fact that these mutations were found in isolates that were circulating in vaccinated birds, coupled with the position of these mutations, is a strong suggestion that suboptimal vaccination may have been the driving force in the selection of these mutants [46], [62]–[64].

In addition, two of the selected mutations found in the CVI isolates (R189K and M226I) were also found in HPAI H5N1 isolates circulating in Pakistan during the 2006–2008 outbreak, indicating increased genetic diversity of Pakistani isolates during that period [65].

Finally, the same A238T mutation we found in the CVI isolates (as well as mutations at positions 140, 189 and 194) have been identified in virus isolates circulating in poultry in China from 2007–2009 [66].

**Figure 4:**
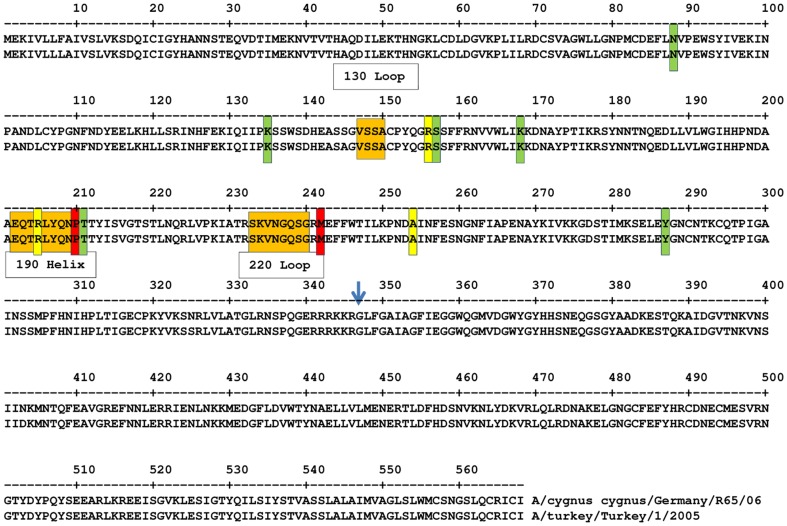
Comparison of positions of HA amino acid mutations found in CVI P42 and FLI P30esc. Positions of mutations in the HA protein found in CVI P42 (yellow bars) and FLI P30esc isolates (green bars) relative to the HA protein sequences of A/turkey/Turkey/1/2005 and A/Cygnus cygnus/Germany/R65/2006. Red bars indicate the two amino acid mutations (P210S, M242I) found in common between CVI P42and FLI P30esc isolates. The positions of the 130- and 220-loop and 190 helix are shown in orange boxes. The blue arrow indicates the proteolytic cleavage site between HA1 and HA2. Numbering according to the A/turkey/Turkey/1/05.

Literature suggests that mutations in or around the 130, 220 loops and the 190 α-helix play a significant role in receptor-type switching [38], [46]. As such, it is not surprising that one of the mutations reported here (M226I) as well as a second mutation not similar to our isolate's but at the same position (R140K), were found in other studies to be associated with an increased affinity for human SA α2,6Gal receptors, thus possibly making these amino acid positions a key to potential avian-to-human transmission [8], [67]. Figure 4 shows the positions of our mutations relevant to these loops. Although substitutions in proteins other than the HA may be necessary before an avian strain gains full pandemic status, the HA amino acid substitutions that result in a change in the affinity from avian to human receptors may be used as markers for vigilance against H5N1 strains that are capable of replicating in humans [67]. Therefore, it is worth studying if any of the mutants reported here show any increased binding affinity to human SA α2,6Gal receptors, as well as whether there is a change in the avian SA α2,3Gal receptors binding affinity of the mutants compared to the parent strains.

Mutation R189K in the HA of an Oseltamivir-resistant human H1N1 virus (A/Tottori/52/08) was found to be critical to viruses with an Oseltamivir-resistant NA 274Y for achieving viral titres similar to that of viruses carrying an Oseltamivir-sensitive NA 274H. This increases the implications of mutations in this particular region of the HA [68]. The same mutation has also been found in isolates from patients affected by the human H1N1 2009 pandemic virus in Portugal, Italy, Japan, Canada and Taiwan among other places [69]–[74]. Mutation R189K has also been found in the consensus HA1 sequences of avian and human H5N1 viruses (Hu, 2010). Literature suggests that another one of the mutations found in both the CVI and FLI isolates (P194S) may also be associated with selection due to Oseltamivir pressure and decreased binding to SA α2,6 receptors probably due to the acquisition of a potential glycosylation site [75]. Indeed, our results demonstrate that this mutation did result in an additional potential glycosylation site as shown in Figure 4. An increase in the number of glycosylation sites has been known to occur at high frequency in the early stages of evolution and may play a role in host adaptation [76]. Additionally, a study by Igarashi [77] suggests that H5 avian influenza viruses are able to acquire N-glycosylation sequons more rapidly than other HA subtypes and other past pandemic viruses.

Antigenic characterisation of the CVI mutants by means of antigenic cartography showed that the antigenic distance between them and the parent strain H5N1 t/T increases with passage number. However, our maps also seem to indicate a diminishing rate of return with increasing passage number. Taken together these results seem to support our previous notion that most of the selection takes place early in the rounds of selection. The same holds true for the distances observed between the FLI isolates and their parent strain H5N1 R65. Our antigenic maps further demonstrate the very large distance between H5N1 t/T or H5N1 R65 and the two reference strains (H5N2 and H5N3). As expected, these distances are much larger than the distance between the parent strains and their mutants. Comparable results were found when comparing the antigenic distances of sera originating from each of the parent strains and mutants mentioned above. It is worth mentioning that the main advantages in the construction of our maps are that they maintain true antigenic distances and avoid the distortion that would result when mapping a 16-dimensional space unto a 2-dimensional one.

The fact that most of the mutations found in the CVI isolates have also been found in chicken in nature is encouraging since it gives credence to our selection procedure as being a valid approach to studying selection of avian influenza mutants. This suggests that our selection method may have relevance to field situations.

It remains to be investigated whether the antigenic distance between our isolates and parent strain is sufficient to allow transmission of the isolates in animals vaccinated against the parent strain or *vice versa*, thus allowing us to really name these isolates escape mutants. Depending on the outcome of such studies, appropriate recommendations regarding vaccine updates can be made.

## Supporting Information

File S1 **Supplemental Tables S1–S5 and Supplemental Figures S1–S2.**
(PDF)
