# Supplementary material for: Immune Escape Mutants of Highly Pathogenic Avian Influenza H5N1 Selected Using Polyclonal Sera: Identification of Key Amino Acids in the HA Protein
Source: PLoS One. 2014 Feb 25;9(2):e84628. doi: 10.1371/journal.pone.0084628 (PMC3934824; doi:10.1371/journal.pone.0084628)
Supplement: File S1 — Supplemental Tables S1–S5 and Supplemental Figures S1–S2. (PDF) [file pone.0084628.s001.pdf]

Supplementary Material

Passage No.	Serum Dilution
1-20	1:400
21-22	1:50
23-24	1:25
25-27	1:10
28-42	1:5

Supplemental Table S1: Overview of homologous and non-immune (control) serum dilutions used for each round of selection. Dilutions were made in DPBS.

Strains	H5N1 t/T	CVI P20	CVI P42	R65	FLI P18esc	FLI P30esc	H5N2	H5N3
H5N1 t/T	0							
CVI P20	1.402480	0						
CVI P42	2.167940	1.533610	0					
H5N1 R65	0.843603	1.694790	2.311480	0				
FLI P18esc	2.610800	3.110060	3.625840	2.640460	0			
FLI P30esc	3.216780	2.702870	2.879320	3.296840	2.777180	0		
H5N2	6.172230	5.901950	6.317610	6.337730	6.667970	6.627570	0	
H5N3	5.291930	5.742920	5.662830	5.418440	4.763730	5.114160	6.081690	0

Supplemental Table S2: Virus distance matrix used for construction of map of strains. Distances are $\log_2$ values. Matrix is symmetrical.

Sera	H5N1 t/T	CVI P20	CVI P42	R65	FLI P18esc	FLI P30esc	H5N2	H5N3
H5N1 t/T	0							
CVI P20	1.44070	0						
CVI P42	1.79865	2.13914	0					
H5N1 R65	1.56097	2.48169	2.09263	0				
FLI P18esc	1.90895	1.80368	3.18838	2.95361	0			
FLI P30esc	1.89271	1.25566	2.44028	3.16373	1.78403	0		
H5N2	4.58370	4.79971	4.32421	3.71186	4.67770	5.11760	0	
H5N3	4.06541	4.58956	4.60298	4.14026	3.66741	4.28147	3.63691	0

Supplemental Table S3: Sera distance matrix used for construction of map of sera. Distances are $\log_2$ values. Matrix is symmetrical.

Sera	Vaccine Groups	Animal Numbers	CVI Strains									
			**H5N1 t/T**	**P5**	**P10**	**P15**	**P20**	**P25**	**P30**	**P35**	**P40**	**P42**
**Sera**	**H5N1 t/T**	376	256	256	256	128	128	256	256	128	128	256
			128	256	128	128	128	256	256	256	128	512
		377	1024	2048	1024	1024	512	512	512	256	256	1024
			1024	1024	1024	1024	512	512	512	512	256	1024
	**CVI P20**	378	2048	1024	1024	1024	2048	512	1024	512	512	1024
			1024	1024	1024	512	1024	512	1024	512	512	1024
		379	512	512	256	512	256	512	512	256	256	512
			256	1024	256	512	256	512	512	256	256	512
	**CVI P42**	742	512	128	128	128	256	1024	1024	1024	1024	1024
			256	128	128	128	512	1024	1024	1024	1024	1024
		743	512	256	512	512	512	1024	2048	2048	1024	1024
			256	256	512	256	256	2048	2048	2048	1024	1024
	**H5N1 R65**	380	512	512	256	256	256	128	256	128	128	256
			512	512	512	256	128	128	256	128	128	256
		381	256	512	256	256	256	256	256	128	128	256
			256	512	512	256	256	256	256	128	128	256
	**FLI P18esc**	382	128	128	128	128	64	128	128	128	128	128
			128	128	128	128	64	128	128	128	128	128
		383	512	512	512	512	256	512	512	256	128	256
			256	512	512	512	256	512	512	256	128	256
	**FLI P30esc**	384	64	32	32	32	32	64	64	64	64	64
			32	32	32	32	64	64	64	64	64	128
		385	128	64	64	64	64	128	128	128	128	256
			128	64	64	64	128	128	128	128	128	256
	**H5N2**	386	64	64	64	64	16	32	32	32	32	32
			64	64	64	64	32	32	32	32	32	32
		387	64	64	32	32	64	64	64	32	32	64
			64	64	32	32	64	64	64	32	32	64
	**H5N3**	744	512	256	256	256	128	64	128	64	64	128
			512	256	256	256	128	128	128	128	64	128
		745	64	64	32	32	32	64	64	64	32	128
			64	64	32	32	32	64	64	64	32	128

Supplemental Table S4: HI data on CVI strains (5 passage intervals) against 16 sera (2 sera/strain). All measurements performed in duplicate.

Strains	Strains									
	H5N1 t/T	CVI P5	CVI P10	CVI P15	CVI P20	CVI P25	CVI P30	CVI P35	CVI P40	CVI P42
H5N1 t/T	0									
CVI P5	1.397620	0								
CVI P10	1.330800	0.977656	0							
CVI P15	1.406130	1.095330	0.504008	0						
CVI P20	1.400300	2.051180	1.966030	1.977350	0					
CVI P25	2.502730	2.952360	2.665530	2.606810	1.733740	0				
CVI P30	2.036800	2.626340	2.354230	2.383640	1.270910	0.765175	0			
CVI P35	2.490260	3.116430	2.758890	2.757260	1.937560	0.871722	1.001720	0		
CVI P40	2.614810	3.294640	2.910200	2.919210	1.945240	1.231290	1.234760	0.752349	0	
CVI P42	2.165900	2.766340	2.603220	2.614870	1.533320	1.216450	1.039340	1.131160	1.381460	0

Supplemental Table S5: Virus distance matrix of CVI isolates from every 5th passage interval. Distances are log_2 values. Matrix is symmetrical.

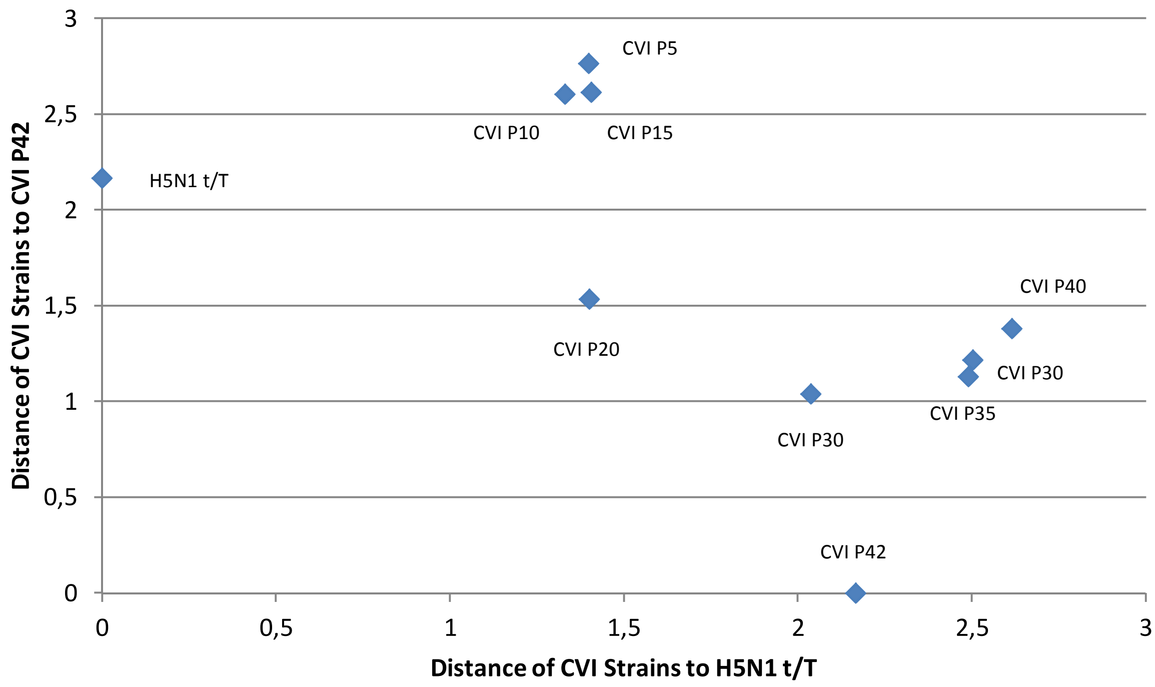

Supplemental Figure S1: Antigenic map showing true distances (log_2) of all CVI isolates in relationship to both H5N1 t/T and CVI P42. CVI P42 is the most distant isolate compared to all other CVI isolates.

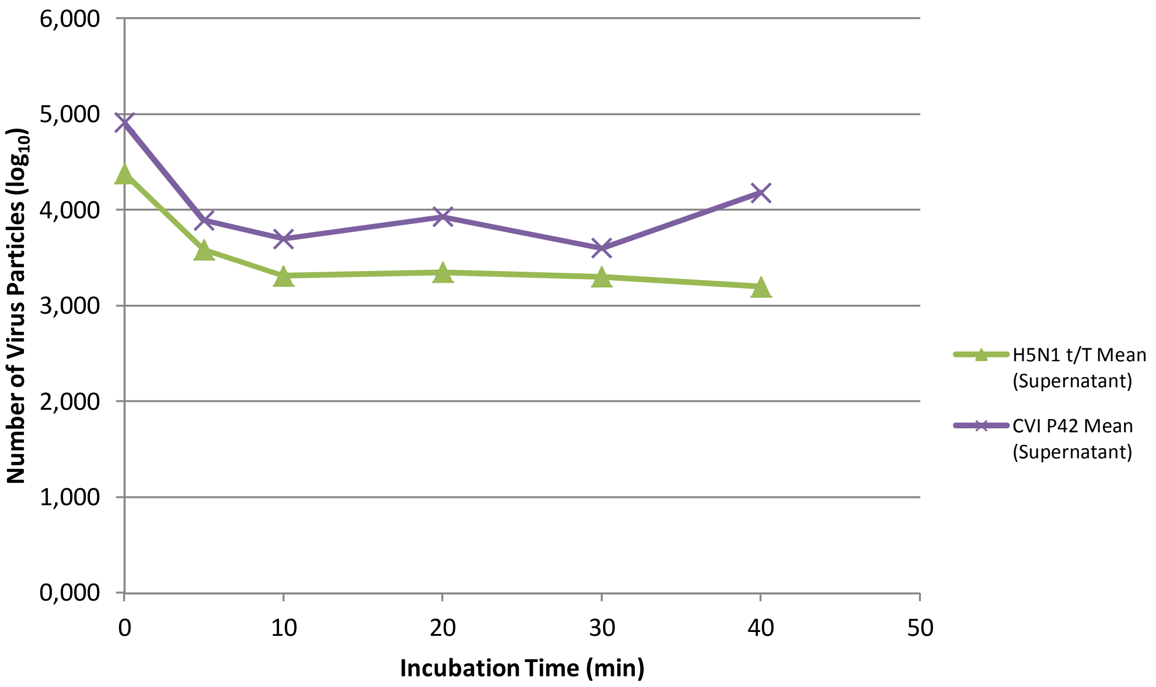

Supplemental Figure S2: Reduction in the number of virus particles in 200µL supernatant, as measured by qPCR at different time points of incubation of virus and 1% chicken erythrocytes on ice. Time point 0min represents virus incubated with DPBS (control).
